# Epidemiology and Risk Factors of Melanoma: A Review

**DOI:** 10.5826/dpc.11S1a161S

**Published:** 2021-07-01

**Authors:** Claudio Conforti, Iris Zalaudek

**Affiliations:** Dermatology Clinic of Trieste, Maggiore Hospital, Piazza Ospitale 1, Trieste, Italy

**Keywords:** Melanoma, epidemiology, risk factors, nevi

## Abstract

We are currently witnessing a worldwide increase in the incidence of melanoma. Incidence in Europe is about 25 cases per 100,000 population, while in Australia it reaches a rate of 60 new cases per 100,000. While the epidemiological curves of the 1980’s and 1990’s suggested an increase in the incidence of melanoma across all age groups, the last 10 years’ data indicates a 5% reduction in the incidence of thin melanoma in young individuals aged between 15 and 24. This suggests a positive impact of primary prevention campaigns [1–2]. The risk factors associated with melanoma are different and multifactorial: on one hand there is a genetic predisposition, as evidenced by the increased risk in patients with dysplastic nevus syndrome, with familial melanoma or familial melanoma syndromes; on the other hand, the unprotected interaction between UV rays and phototypes I–II increases the risk of developing melanoma, especially in case of sunburns in pediatric age. This review aims to summarize melanoma epidemiology and risk factors.

## Introduction

Melanoma incidence is increasing in white skin populations, especially where fair-skinned individuals are subject to excessive sun exposure, as attested by incidence and prevalence data of skin cancers in Australia [3]. In Europe, the incidence rate is about <25 new cases of melanoma per 100,000 population; in the United States (US), 30 per 100,000; and in Australia, where the incidence rate is extremely high, it reaches 60 cases per 100,000. In recent years, there has been a dramatic increase in incidence in people over 60 years of age and in general in all age groups. Incidence curves suggest that the incidence will continue to increase in the coming years [4]. The most common phenotypic risk factor is sunburn-prone skin, whereas melanocortin-1 receptor (MC1R) gene variants are the most important underlying genetic determinants studied in the last decade. Individuals with a high number of common nevi and those with large congenital, multiple, and/or atypical nevi are at higher risk, and this phenotype is also genetically determined [5]. Melanoma is more likely to be diagnosed in these groups of patients, which is why they need thorough follow-up and clinical monitoring. The genetic component is part of the increased risk although it is not the main factor. The most important exogenous factor of melanoma is UV exposure, particularly intermittent sun exposure [6].

### Epidemiology and Risk Factors of Melanoma

#### Incidence Trends

Cutaneous melanoma (CM) is by far the most common subtype of melanoma, accounting for more than 90% of melanoma cases [3].

Since the Second World War, the incidence of CM has increased while Australian and North American data showed a stabilization of CM rates [7].

In these countries, primary and secondary prevention campaigns have increased allowing to limit the damage mediated by UV rays. This has been obtained by increasing information diffusion on the importance of sun protection, by implementing diagnostic systems, such as dermoscopy, allowing for early suspicious diagnosis.

Queensland (Australia) epidemiological trends during the last 10 years, show a 5% decrease of thin melanoma incidence in young individuals between 15 and 24 years, suggesting that primary prevention efforts are being carried out successfully [7–8]. On the other hand, a significant reduction in mortality in all age groups has not yet been observed [8].

Melanoma is reported as the 19th most common cancer worldwide, with estimated age-adjusted incidence rates of 2.8–3.1 per 100,000 [9].

The analysis of CM trends in Europe between 1995 and 2012, shows an incidence rate ranging from 5.6/100,000 inhabitants in Spain to 24/100,000 in Switzerland where there is the highest number of diagnosed in situ melanomas [7].

The median age at diagnosis is 61 years for men, 56 for women. In situ melanomas constituted 25% of diagnosed melanomas, while superficial spreading melanoma (SSM) was the most frequent variant constituting 46% of diagnoses. As regards lesions’ distribution, this varied between men and women: in men the most frequent site was the trunk (43%), in women the legs (57%) [7].

Otherwise, Australia and the US have higher incidence rates compared to Europe. The reason for this marked incidence variation is unclear and could be associated with cultural and wealth differences influencing the sun exposure time. Another reason could be due to the fact that many European countries do not have a cancer registry, or this is not rigorously updated [6].

The incidence of melanoma is increasing at a greater rate than other types of cancer. The mean age at diagnosis is 57 years with higher incidence in women in the younger age groups while the ratio reverses in old age with higher incidence in men. Estimates from the US report a lifetime risk of melanoma of 1 in 56 for women and 1 in 37 for men. In general, mortality rates are higher among men than women [5,6], possibly due to the later presentation of the disease.

### Risk Factors: Photo-Type, Nevus Count, Ultraviolet Rays

Several risk factors thought to be significant in the development of cutaneous melanoma have been identified by epidemiologic studies. These can be divided into environmental factors and genetic factors, but there is clearly an interaction between genetics and environment.

Pigmentation has an indisputable and significant influence on skin susceptibility to malignant change. Melanocortin 1 receptor (MC1R) is a cell surface receptor in melanocytes that induces pigment production. There are many polymorphisms of *MC1R* gene, which determine the different skin phenotypes; variants such as red hair and fair skin phenotype express low pigmentation, resulting in increased sensitivity to ultraviolet (UV) light and an increased risk of associated melanoma.

In addition to characterizing the phototype, melanin, is involved in defending melanocytes and keratinocytes from UV light; this explains why phototypes I and II are at higher risk of developing melanocytic and keratinocytic cancers, being more susceptible to UV damage.

A high number of acquired melanocytic nevi, the red hair phenotype and MC1R R alleles all independently increase melanoma risk.

This is supported by a study carried out in Queensland, Australia, reporting that individuals with ≥ 20 nevi (≥ 5 mm diameter) and MC1R R/R genotype have a 25-fold increased melanoma risk, compared to people with 0 to 4 nevi and the MC1R WT/WT genotype; while individuals with ≥ 20 nevi and the MC1R R/R genotype have an absolute melanoma risk to age 75 of 23,3% for men and 19, 3% for women [10].

Several studies have shown that the main factors associated with the development of melanoma are the number of melanocytic nevi, family history of melanoma, and genetic susceptibility. Melanoma in most cases arises on healthy skin, although 25% of melanomas are associated with a preexisting nevus and this justifies the double incidence of nevus associated melanoma in young adults and elderly. In addition, the number of moles is associated with the risk of developing melanoma especially in cases of more than 100 moles or moles with dysplastic appearance [11].

Most cutaneous melanomas arise on skin sporadically (rather than chronically) exposed to the sun, in sites and individuals who are more prone to sunburn. The highest rates are seen in individuals with repeated intense sun exposure. This theory is further strengthened by the observation that patients with melanoma who actively reduce their sun exposure after initial diagnosis are consequently at reduced risk of developing a second primary melanoma [8]. In contrast, individuals with dark skin, or skin that darkens easily in response to sunlight but does not burn, have demonstrably lower rates of melanoma [12–13]. However, sun exposure is not directly related to melanoma development, as evidenced by the fact that melanoma can also occur in sites that are not chronically exposed to the sun.

The age at which sun exposure and/or sunburn occurs also appears to be important. A systematic review [14] strongly associated intermittent sun exposure in childhood or adolescence with an increased risk of melanoma. Specifically, individuals who experienced more than 5 episodes of severe sunburn had a 2-fold increased risk of melanoma [15].

One of the most important modifiable risk factors in the etiopathogenesis of melanoma is certainly UV-B exposure [16]. Personal history of sunburn in childhood is associated with a higher risk, intermittent exposure is associated with melanoma, and chronic exposure is associated with actinic keratosis and keratinocyte cancers.

Although the melanoma-effects of UV-B exposure are well evidenced, UV-A exposure does not come without risks [17].

Artificial UV exposure may play a role in the development of melanoma; in fact, the amount of UV-A exposure in a typical tanning bed session is significantly higher than exposure during normal outdoor activities or even during sunbathing

Sunbeds emit UV-A radiation and a meta-analysis of studies [18] that explored the incidence of melanoma following sunbed use reported a 75% increased risk in individuals under 35 years of age with a history of sunbed use. Because of the increased risk of melanoma in tanning bed users, their use has been banned in many states [19]. Instead, smoking, a common carcinogen, has not been independently associated with melanoma [20].

Finally, there is an interesting association between melanoma and comorbidities. For instance, immunosuppressed individuals, who underwent organ transplantation, are at demonstrable risk for melanoma, including recurrence in individuals with primary melanomas resected before transplantation, although the greatest risk for these patients is to develop keratinocyte cancers. In fact, the pooled relative risk (pRR) for melanoma, among liver and heart transplant patients was 5.27 (95% CI 4.50–6.62), higher than the pRR in kidney transplant patients 2.54 (95% CI 2.18–2.96). According to recent data, transplant recipients are at more than double the risk of developing melanoma overall when compared to the general population [21].

In addition, patients who present other skin malignancies (basal cell or squamous cell carcinomas or mycosis fungoides) are at higher risk of developing melanoma and subsequent death from the disease [22,23].

### Genetic Factors

A family history of melanoma is a strong risk factor for the disease. Considering that familial clustering of a disease is an indicator of possible heritable causes, there has been an explosion of research in the past 2 decades directed at elucidating the genetic basis of melanoma [24].

This explains why it is important to also consider the individual genetics when determining personal risk. Genetic factors such as skin phenotype, clearly influence risk, as well as familiarity that counts for 5–10% of melanomas origin [25].

Some of these occur in specific syndromes-such as atypical familial multiple moles and melanoma syndrome (FAMMM) or dysplastic nevus syndrome (DNS)-where individuals have multiple, phenotypically variable moles at high risk for malignant transformation, thus presenting an almost guaranteed lifetime risk of melanoma. Many individuals do not meet the diagnostic criteria for these syndromes, but still have numerous nevi, often due to cumulative sun exposure [25].

Observational studies suggest a strong association between a high number of nevi and melanoma [26]. A personal history of cutaneous melanoma is also a known risk factor for additional primary melanomas [27]. All of these criteria are of great clinical value because patients with so many nevi, with familiarity for melanoma, and with dysplastic nevus syndrome are monitored nowadays with digital videodermatoscopy by performing quarterly or semiannual total body dermatoscopic examination.

To assess individual risk and to carry out successfully prevention interventions risk prediction models have been developed in recent years. Clinicians and patients have now access to a series of online calculators assisting in prevention stages, early detection, and optimum treatment of melanoma, ultimately saving lives [28]. There are several variables considered in these scores, the most common being the presence of moles, freckle density, history of sunburns, and hair color [29].

### Mucosal Melanoma

Mucosal melanoma is the least common of the 3 melanoma subtypes, accounting for less than 1.5% of all melanomas [30]. The incidence of mucosal melanoma varies with both gender and age [30], the median age at diagnosis is 70, except for oral cavity melanomas which tend to occur in younger patients. Incidence increases with age, over 65% of cases are in fact diagnosed in patients over 60. The incidence in women is almost twice as high as in men, possibly because of the higher rates of genital tract melanomas [31] amongst women. The absolute incidence of mucosal melanoma in white populations is higher (2:1) than in non-whites [30–33].

Mucosal melanomas occur most often in the head and neck region, the female genital tract, and the anorectal region [30]. No clear risk factors for mucosal melanoma are known. Because mucous membranes are not exposed to the sun, UV radiation is not considered an important etiologic factor. The role of viruses-such as human papillomavirus (HPV) or human herpes virus (HHV) implicated in other malignancies of the oral cavity-has not been demonstrated [32] while smoking has been reported to be associated with a higher prevalence of oral pigmented lesions [34].

## Conclusion

Worldwide data indicates an increase in the incidence of melanoma, although primary and secondary prevention campaigns have led to a 5% reduction in the incidence of thin melanoma in individuals between 15–24 years of age, suggesting the effectiveness of preventive measures [1–2].

While it is possible to intervene with early diagnosis, there are non-modifiable risk factors that must be evaluated for each patient. Among these, photo type, number of nevi, familiarity for melanoma are independent variables associated to melanoma.

It is therefore necessary to intervene on the removal of known risk factors such as avoiding sunburn, avoiding the use of tanning lamps, and exposing to the sun without using sunscreen.

A recent study showed that broad-spectrum sunscreens that prevent erythema are unlikely to compromise vitamin D status in healthy populations. This explains the futility of avoiding sunscreen to produce Vitamin D, a theory often expressed by patients who fear the side effects of sunscreen. Based on these data, a daily broad-spectrum sunscreen with high UV-A protection does not compromise vitamin D status in healthy people and should always be used, regardless of the season [35].

On the other hand, it is necessary to implement screening campaigns even in younger age groups and carry out informative campaigns to stress the importance of an annual dermatological checkup. Moreover, to improve secondary prevention it is essential to disclose easy rules, such as the ABCDE rule (asymmetry, irregular borders, uneven color, size greater than 6 mm, and history of evolution) or the ugly duckling (a mole different from the others) that are still effective today for the early diagnosis of melanoma.

The development of new technologies, such as dermoscopy, videodermoscopy and confocal microscopy, have also increased the diagnostic capacity in small melanocytic lesions [36, 37].

This fact allows earlier diagnoses, but it increases the diagnostic capacity and therefore the incidence of the disease. From 1975 to 2015, the incidence of melanoma increased approximately 6-fold in the US. The cause of this increase, according to some authors, is not due to UV-induced sun damage or to personal risk factors but to an increased clinical and histological ability to diagnose melanoma [38,39]. In fact, according to some studies, although the incidence of melanoma has increased in most continents, mortality has remained stable. A recent work points out that the cause of the increase in diagnosis and therefore incidence of melanoma is due to a medical-legal problem, ie, more dermatologists perform biopsy analysis on suspicious lesions and more pathologists tend to diagnose melanoma, even when they are faced with “gray spaces” as in the case of dysplastic nevi [38]. According to the authors, this cycle of overdiagnosis is intensified by the use of the dermatoscope by dermatologists, which increases the number of lesions excised. The authors’ suggested solution to limit overdiagnosis would be to stop mass dermatological screening.

The data reported in our review confirms an increase in the incidence of melanoma, although mortality remains stable. However, primary and secondary prevention campaigns have had a positive impact in reducing the diagnosis of melanoma, particularly in younger populations, as previously reported and discussed. Moreover, dermoscopy, has not only increased the number of removed melanomas, it has also allowed to avoid benign lesions’ removal. On this point we disagree with the authors who claim that screening campaigns should be suspended because an annual examination allows the early detection of melanomas and the identification of high-risk patients (eg patients with more than 100 nevi or with dysplastic nevus syndrome) who need a closer monitoring. Future studies are needed to identify the effectiveness of primary and secondary prevention, to assess its impact on worldwide incidence, and to solve current controversies.
